# The mediating effect of nutrition on oral frailty and fall risk in community-dwelling elderly people

**DOI:** 10.1186/s12877-024-04889-3

**Published:** 2024-03-20

**Authors:** Huizi Song, Yulian Wei, Yan Wang, Jiahui Zhang

**Affiliations:** 1 School of Nursing, School of Public Health, Yangzhou University, Yangzhou, Jiangsu China; 2 Department of Geriatrics, Northern Jiangsu Peoples Hospital, Yangzhou, Jiangsu Province China; 3 Taizhou People’s Hospital, Jiangsu, Taizhou China

**Keywords:** Oral frailty, Nutrition, Fall risk, Community elderly, Mediation analysis

## Abstract

**Background:**

Population aging is accelerating, particularly in Asian countries. Falls are the leading cause of unintentional injuries in the elderly over 60 years old in China. Hence, it is crucial to anticipate the risk factors associated with fall risk. We aimed to explore whether oral frailty and fall risk were reciprocally related and whether nutrition mediated their association.

**Methods:**

From October 2022 to March 2023, a total of 409 elderly individuals from the Yangzhou community were selected using the convenience sampling method. Cross-sectional data on older adults’ oral frailty, nutrition, and fall risk were collected using questionnaires. Data analysis was performed using SPSS 27.0 and PROCESS macro.

**Results:**

The fall risk score was 1.0 (ranging from 0 to 4.0), with 107 cases (26.2%) identified as being at risk of falling. Spearman correlation analysis revealed a positive correlation between oral frailty and the risk of falls (*rs* = 0.430, *P* < 0.01). Nutrition was found to have a negative correlation with both oral frailty and fall risk (*rs*=-0.519、-0.457, *P* < 0.01). When controlling for covariates, it was observed that nutrition mediated the relationship between oral frailty and falls. The mediating effect value accounted for 48.8% of the total effect (*P* < 0.01).

**Conclusions:**

Oral frailty was significantly associated with fall risk, and nutrition might be a mediating factor for adverse effects of oral frailty and fall risk. Enhancing the nutrition of older individuals is a vital approach to mitigating fall risk among those with oral frailty.

## Introduction

Globally, the number of people aged 65 years was 703 million in 2019 [1]. Moreover, the aging population rate is accelerating, especially in Asian countries. According to the bulletin of the 7th National Population Census, the proportion of individuals aged 60 and above in China is 18.3% [2]. This represents a 5.44% increase in the proportion of elderly people compared to the data from the 6th National Population Census. China is experiencing a transition from mild to moderate aging, with the proportion of the elderly population projected to reach approximately 25% by 2030 [3]. According to the World Health Organization, about 28-35% of the elderly over 65 years old fall each year, of which 4-15% will cause major injury [4]. Studies have reported that falls are the first cause of unintentional injuries in the elderly over 60 years old in China, accounting for about 52.81% of the total number [5]. The disease burden of falls in China is about twice that of the United States [6]. Falls in older people can have serious consequences, including death, disability, and a decline in daily functioning. This can result in higher healthcare costs, and increased need for medical care, rehabilitation, and support services, and can also place a burden on families and society [7]. Therefore, predicting the risk factors for falls and developing strategies to minimize them in the elderly populations are the main concerns of clinicians and policymakers in aged societies.

Oral frailty, a geriatric syndrome, refers to a decline in oral function accompanied by a decline in cognitive and physical functions [8]. It is estimated that the incidence of oral weakness in the elderly ranges from 8.4–22.7% [9], which significantly impacts their quality of life. Poor oral health can result in increased physical frailty, disability, and mortality rate [10–13]. Studies have pointed to the impact of poor oral conditions and occlusal support on postural stability and balance function in the elderly, which may lead to fall-related events [12]. Previous studies have identified potential factors contributing to the heightened risk of falls in elderly individuals with oral frailty. However, there remains a need for more precise data and comprehensive analysis to explore the specific relationship between them.

Malnutrition, which is the most prevalent health issue among the elderly, is characterized by insufficient nutrient intake that results in impaired physical and mental functioning, ultimately affecting clinical outcomes [14]. It is worth noting that malnutrition plays a crucial role in sarcopenia, significantly influencing its functionality, independence, and overall quality of life [15–17]. Extensive research has demonstrated that elderly individuals with inadequate nutrition face a greater risk of falling [18, 19]. In addition, there is a relationship between nutrition and oral health. Oral weakness can decrease the elderly’s willingness to eat independently, leading to inadequate nutrition intake and an increased risk of malnutrition [20]. While previous studies have indicated a connection between oral frailty, nutrition, and fall risk, no specific mechanisms have been reported.

The purpose of this study is to investigate the relationship between oral frailty, nutrition, and fall risk among elderly people in the community. Additionally, the study aims to explore whether nutrition plays a mediating role between oral frailty and fall risk. Understanding the interrelationship between these factors will inform healthcare providers in developing interventions to reduce poor health outcomes caused by falls in older adults. This study aims to provide a reference for interventions aimed at preventing falls among the elderly.

## Materials and methods

### Participants

This is a cross-sectional study and participants were recruited from their communities in Yangzhou City using convenience sampling. The inclusion criteria are: (1) age ≥ 60 years; (2) live in the community for at least 6 months; (3)individuals who are capable of independently completing the questionnaire or can do so with the assistance of a researcher. The exclusion criteria are (1) the participants were diagnosed with severe mental disorders and dementia by professional medical institutions; (2) those with cognitive impairment and unable to complete the questionnaire; (3) the elderly in the acute or terminal stage of the disease; (4) severe visual or hearing impairment. All participants voluntarily participated in the study and signed an informed consent form.

### Measurements

#### Demographic characteristics

Demographic characteristics, including age, sex, race, education level, place of residence, monthly income, and marital status, were collected. Lifestyle characteristics, such as drinking alcohol history and smoking history, were also recorded. Participants self-reported any chronic diseases they had and the type of prescription medications they were taking. Chronic diseases include chronic respiratory diseases, diabetes mellitus, hyperlipidemia, coronary heart disease, hypertension, stroke, chronic gastritis, gastric ulcer, osteoporosis, and lumbar/cervical spondylosis. History of smoking or drinking was defined as daily smoking or drinking for more than three months.

#### Oral frailty index-8

The Oral Frailty Index-8(OFI-8) was developed by the Japanese Dental Association. The scale consists of eight items, which are as follows: (1) Are you unable to eat hard and dry food for more than six months? (2) Have you choked on your tea/soup? (3) Do you use dentures? (4) Do you have dry mouth symptoms? (5) Have you gone out less than six months ago? (6) Can you chew hard and dry food? (7) Do you brush your teeth at least twice a day? (8) Do you visit the dentist at least once a year? Items (1) to (3) are each worth 2 points, while the other items are each worth 1 point, resulting in a total score of 11 points. Scores ranging from 0 to 2 are classified as low risk, 3 as medium risk, and 4 or more as high risk. The Cronbach’s *α* coefficient of the scale was 0.692 and both the sensitivity and specificity were 80% [21].

#### Short form mini nutritional assessment

The Short-form mini nutritional assessment(MNA-SF) proposed by Rubenstein in 2001, based on the mini nutritional assessment (MNA), is used to assess the nutritional status of the elderly [22]. It consists of 6 items: BMI, recent body mass loss, acute illness or stress, bedridden status, dementia or depression, decreased appetite, or difficulty eating. The scale has a total of 14 points, with a score of less than 11 indicating the risk of malnutrition, and a score of 11 or more indicating good nutritional status.

#### Stay independent brochure questionnaire

The Stay Independent Brochure (SIB) was issued by the U.S. CDC and underwent sinicization and reliability testing by Chinese scholar Li Yaling [23]. SIB is a widely used fall-risk self-assessment tool, which is part of the Stopping Elderly Accident, Deaths & Injuries (STEADI) program in the US. The scale comprises 12 entries, which are answered with either ‘yes’ or ‘no’. The scoring system assigned 2 points for a ‘yes’ response to the first two items on the scale, and 0 points for a ‘no’ response. For the last 10 items, a ‘yes’ response was given 1 point, while a ‘no’ response received 0 points. The scale’s total score ranges from 0 to 14, with a score of 4 or higher considered to be at risk for falls. The scale exhibited good reliability and validity, with a Cronbach’s *α* coefficient ranging from 0.608 to 0.716 and a content validity index of 0.920. Therefore, it can be used for independent assessment of fall risk among elderly individuals in communities [24].

#### Oral health assessment tool

This Oral Health Assessment Tool(OHAT) has been revised and developed by Chalmers et al. in 2015 [25]. The assessment evaluated eight aspects related to oral health: lips, tongue, gingival tissue, saliva, natural teeth, dentures, oral cleanliness, and toothache. Each aspect was assigned a score of 0 (indicating normal), 1 (indicating diseased), or 2 (indicating abnormal). The total score ranged from 0 to 16, with higher scores indicating poorer oral health. The Cronbach’s *α* coefficient of the scale was 0.780.

#### Tilburg frailty index

The Tilburg frailty index(TFI) was developed by Gobbens et al. for self-rated frailty status in the elderly [26]. It consists of three dimensions: physical frailty (including walking ability, balance ability, hearing, vision, grip strength, fatigue, physical health, and weight loss), psychological frailty (including memory, depression, anxiety, and problem-handling ability), and social frailty (including living alone, social connection, and social support). The scale comprises a total of 15 items, which were scored using a two-category method, where 1 point was given for each symptom. The total score ranged from 0 to 15, with higher scores indicating greater severity of frailty. The Cronbach’s *α* coefficient of the scale was 0.710.

### Data collection methods

This study was conducted by the Declaration of Helsinki and approved by the Clinical Research Ethics Committee of the Yangzhou University School of Nursing School of Public Health (YZUHL20220046). Eligible patients are invited to participate in the study and are informed of the purpose and process of the study. After obtaining written consent from the patients, the researchers distributed paper questionnaires to senior citizens in the community and conducted laboratory tests. The patients had the option to independently fill out the questionnaire or have the researcher read the questions aloud to assist them. In cases where the patients did not understand the medical terms used in the questionnaire, the researcher provided a brief explanation. The researcher collected the questionnaires on-site and checked for any errors or omissions, assisting the patients in correcting or filling in any missing information. A total of 446 questionnaires were sent out in this study, out of which 409 were considered valid, resulting in an effective recovery rate of 91.7%.

### Data analysis

This study used SPSS 27.0 (version 27.0 Chicago, IL, USA) to conduct statistical analysis. Firstly, we compared the demographic characteristics of fall risk among the participants. Counting data were described by frequency and component ratio. The measurement data that followed a normal distribution were described using means ± standard deviation, while the measurement data that did not follow a normal distribution were described using M(P25, P75). Because the fall risk scores under different categories did not conform to the normal distribution, the Wilcoxon rank sum test was used. Secondly, correlation analysis and linear regression analysis were used to explore the relationship between nutrition, oral frailty, and fall risk. The total score of nutrition and oral frailty followed a normal distribution, while the total score of fall risk did not. Therefore, Spearman rank correlation analysis was conducted to investigate the correlation between oral frailty, nutrition, and fall risk. Finally, Model 4, a simple intermediary model, from the SPSS macro program Process developed by Hayes, was used to analyze the mediating effect between oral frailty, nutrition, and fall risk [27, 28]. All regression coefficients were tested using the bias-corrected percentile bootstrap (repeated sampling 5000 times) while controlling for measured covariates [29]. A bilateral test with a significance level of *α* = 0.05 was employed.

## Results

### Demographic characteristics

The study included 409 community-dwelling elderly individuals, with an average age of 71.89 ± 7.58. Among the participants, 52.6% were female, and 40.1% had completed high school education or above. The majority (84.8%) were married, and 64.8% resided in urban areas. Additionally, 35.7% of the participants were classified as overweight or obese, while 5.4% were underweight. It was found that 26.4% of elderly individuals consumed alcohol, and 20.5% were smokers. In this study, 40.3% of the elderly participants had one chronic disease, while 53.8% had two or more chronic diseases. Additionally, 50.4% of the elderly took 1–3 types of prescription drugs, and 15.6% took four or more types. The mean OHAT score was 3.4 ± 2.7. The mean TFI score was 3.3 ± 2.5, which included physical frailty 1(0,2), mental frailty 1.1 ± 1.1, and social frailty 0.9 ± 0.6.

### Oral frailty, nutrition, and fall risk scores of the elderly in the community

The SIB score ranged from 0 to 4.0, with a mean of 1.0. Out of the total cases, 107(26.2%) were found to have a fall risk. The MNA-SF score had a mean of 10.3 ± 1.9, and 188 (46.0%) were identified as malnourished. The OFI-8 score had a mean of 3.4 ± 2.1, with a total of 155 (37.9%) cases classified as low risk, 85 (20.8%) cases as moderate risk, and 169 (41.3%) cases as high risk.

### Demographic characteristics of fall risk in community-dwelling elderly people

Table 1 demonstrates the presence of statistically significant differences in the risk of falls among the elderly in the community in age, educational level, marital status, place of residence, types of chronic diseases, OFI-8 level, and MNA-SF level(*P* < 0.05).

**Table 1:** Demographic characteristics of fall risk in community-dwelling elderly people(*N* = 409)

Variable	Total *N* = 409	Fall risk Median, (Q1-Q3)	*P*
Gender, N(%)			0.365
Male	194(47.4)	1(0,4)	
Female	215(52.6)	1(0,4)	
Age ground, N (%)			<0.001
60–69	179(43.8)	0(0,2)	
70–79	168(41.1)	1(0,4)	
80+	62(15.2)	4(2,8)	
BMI grading, N (%)			0.329
Underweight	22(5.4)	0(0,3.25)	
Normal weight	241(58.9)	1(0,4)	
Overweight	135(33.0)	1(0,4)	
Obese	11(2.7)	3(1,4)	
Education level, N (%)			0.048
Primary school and below	153(37.4)	2(0,4)	
Junior secondary school	92(22.5)	1(0,3)	
High school/technical secondary school	99(24.2)	1(0,3)	
College and above	65(15.9)	0(0,3)	
Marital status, N (%)			<0.001
Married	347(84.8)	1(0,3)	
Unmarried/divorced/widowed	62(15.2)	3.5(1,7.25)	
Place of residence, N (%)			0.048
Countryside	144(35.2)	2(0,4)	
City	265(64.8)	1(0,3)	
Drinking alcohol history, N (%)			0.234
Yes	108(26.4)	1(0,3)	
No	301(73.6)	1(0,4)	
Smoking history, N (%)			0.812
Yes	84(20.5)	1(0,3)	
No	325(79.5)	1(0,4)	
Monthly income, N (%)			0.099
< 2000 RMB	72(17.6)	2(0,5)	
2000–5000 RMB	145(35.5)	1(0,4)	
> 5000 RMB	192(46.9)	1(0,3)	
Chronic disease status, N (%)			<0.001
0	24(5.9)	0(0,1.75)	
1	165(40.3)	0(0,2)	
≥ 2	220(53.8)	2(0,5)	
Number of prescription drugs, N (%)			0.240
0	139(34.0)	1(0,4)	
1–3	206(50.4)	1(0,4)	
≥ 4	64(15.6)	0(0,3)	
TFI score, Mean (SD)	3.3 ± 2.5		
Physical frailty, Median, (Q1-Q3)	1(0,2)		
Psychological frailty, Mean (SD)	1.1 ± 1.1		
Social frailty, Mean (SD)	0.9 ± 0.6		
OHAT score, Mean (SD)	3.4 ± 2.7		
OFI-8 level, N (%)			<0.001
Low risk	155(37.9)	0(0,2)	
Medium risk	85(20.8)	0(0,2)	
High risk	169(41.3)	3(1,6)	
MNA-SF level, N (%)			<0.001
Malnutrition	181(44.3)	3(1,6)	
Eutrophy	228(55.7)	0(0,2)	

Note. Means ± standard deviation was shown. Data are shown using %, mean (standard deviation) or median (interquartile range). *P* values were calculated with Mann–Whitney-U test. *BMI*: body mass index. TFI: The Tilburg frailty index. OHAT: The Oral Health Assessment Tool. OFI-8: The Oral Frailty Index-8. MNA-SF: The Short Form Mini Nutritional Assessment

### Correlation analysis of oral frailty, nutrition, oral health status, status of frailty, and fall risk scores

Spearman rank correlation analysis revealed a positive correlation between oral frailty, oral health, frailty status score, and fall risk (*rs* = 0.430, 0.361, 0.386, *P* < 0.01). Additionally, there was a negative correlation between nutritional status and both oral weakness (*rs*=-0.519, *P* < 0.01) and fall risk (*rs*=-0.457, *P* < 0.01), as displayed in Table 2.

**Table 2:** Correlation analysis of oral frailty, nutrition, oral health status, status of frailty and fall risk scores(*N* = 409)

Variable	1	2	3	4	5	6	7	8
1. Fall risk	1		—	—	—	—	—	—
2. Nutrition	-0.457**	1	—	—	—	—	—	—
3. Oral frailty	0.430**	-0.519**	1		—	—	—	—
4. Oral health status	0.361**	-0.245**	0.348**	1	—	—	—	—
5. Status of frailty	0.386**	-0.228**	0.325**	0.398**	1	—	—	—
6. Physical frailty	0.378**	-0.219**	0.338**	0.376**	0.844**	1	—	—
7. Psychological frailty	0.103*	-0.081	0.133**	0.138**	0.457**	0.161**	1	—
8. Social frailty	0.351**	-0.211**	0.216**	0.349**	0.717**	0.406**	0.157**	1

Note: — indicates duplicate data; * *P* < 0.05;** *P* < 0.01

### Examination of the mediating effect of nutrition on oral frailty and fall risk

**Fig. 1:**
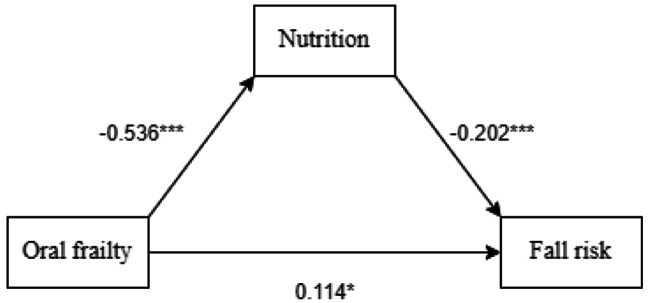
Path diagram of the mediating effect of nutrition on the association between oral frailty and fall risk. *P < 0.05, ***P < 0.001

After controlling for age, education level, marital status, place of residence, types of chronic diseases, Oral health status, and frailty status, we examined the mediating effect of nutrition on the relationship between oral frailty and fall risk. The results showed that oral frailty was a significant predictor of fall risk (*β* = 0.22, *t* = 4.84, *P* < 0.001). Even after accounting for nutrition, the direct prediction effect of oral frailty on fall risk remained significant(*β* = 0.11, *t* = 2.19, *P* = 0.03). Oral frailty was also found to be a significant predictor of nutrition(*β*=-0.54, *t*=-11.46, *P* < 0.001), and nutrition was a significant predictor of fall risk (*β*=-0.20, *t*=-4.19, *P* < 0.001) (see Fig. 1; Table 3). In addition, the upper and lower limits of the bootstrap 95% interval for the direct and mediating effects of the effect of oral frailty on fall risk did not include 0 (see Table 4). This suggests that oral frailty can directly affect fall risk and indirectly affect nutrition through the mediating effect of nutrition. The total effect size was 0.354, the direct effect size was 0.181, and the intermediate effect size was 0.173. Thus, 48.8% of the effect of oral frailty on fall risk was achieved through the mediating effect of nutrition. The mediation effect model is shown in Fig. 1.

**Table 3:** Examination of the mediating effect of nutrition on oral frailty and fall risk(*N* = 409)

Model	Model 1			Model 2			Model 3		
Dependent variable	Fall risk			Nutrition			Fall risk		
Indicators	*β*	*t*	*P*	*β*	*t*	*P*	*β*	*t*	*P*
Oral frailty	0.222	4.837	<0.001	-0.536	-11.464	<0.001	0.114	2.192	0.029
Nutrition							-0.202	-4.191	<0.001
Age ground	0.180	4.031	<0.001	-0.034	-0.744	0.457	0.173	3.955	<0.001
Education level	0.091	2.112	0.035	-0.049	-1.125	0.261	0.081	1.916	0.056
Marital status	0.069	1.645	0.101	-0.028	-0.662	0.509	0.063	1.539	0.125
Place of residence	-0.127	-3.036	0.003	0.154	3.601	<0.001	-0.096	-2.305	0.022
Chronic disease status	0.207	4.950	<0.001	-0.112	-2.639	0.009	0.184	4.459	<0.001
Oral health status	0.096	2.015	0.045	-0.018	-0.375	0.708	0.093	1.977	0.049
Physical frailty	0.131	2.843	0.005	-0.011	-0.236	0.813	0.128	2.852	0.005
Social frailty	-0.010	-0.249	0.804	0.016	0.393	0.694	-0.007	-0.171	0.864
Psychological frailty	0.178	3.861	<0.001	-0.051	-1.085	0.279	0.167	3.707	<0.001
*R* ^*2*^	0.635			0.618			0.654		
Adjusted *R*^*2*^	0.403			0.382			0.428		
*F*	26.834			24.579			27.006		

Note. Model 1: multiple linear regression analysis between fall risk and oral frailty, Model 2: multiple linear regression analysis between nutrition and oral frailty, Model 3: multiple linear regression analysis between fall risk and oral frailty adjusted by nutrition

Adjusted by age, educational level, marital status, place of residence, types of chronic diseases, oral health status, status of frailty.

**Table 4:** Direct and indirect effects of oral frailty on fall risk (*N* = 409)

	Effect	Boot SE	BootLL CI	BootULCI	Proportion of effect
Total effect	0.354	0.073	0.210	0.498	
Direct effect	0.181	0.083	0.019	0.344	51.2%
Indirect effect	0.173	0.062	0.063	0.304	48.8%

Note. SE: standard error. CI: 95% Confidence Interval

## Discussion

The study examined the association between oral frailty and fall risk in adults over 60 years and further revealed the underlying mechanisms by establishing a mediation model. Our study stated that oral frailty was significantly associated with fall risk. Furthermore, nutrition partially mediated the effects of oral frailty on fall risk, with a mediation effect of up to 48.8%. These results suggest that improving nutrition could mitigate the negative impact of oral frailty on fall risk.

The results of this study indicate that 26.2% of individuals aged over 60 in China may be at risk of experiencing a fall. When considering the average annual fall incidence of 14.7–34% among the elderly in China [30], it becomes evident that there is a significant potential for falls despite the already high fall rate. Falls are the primary cause of injury in the elderly population, with 20–30% of fall victims experiencing moderate to severe injuries, such as lacerations, fractures, and traumatic brain injuries [31]. In particular, hip fractures can lead to complications like deep vein thromboembolism and infection, resulting in prolonged hospital stays and increased mortality rates [32]. The financial burden associated with fall-related injuries is substantial, with costs ranging from $16 per person per incident to $3,812 per person per incident in China [33].

Previous studies have reported that fall risk is influenced by various demographic factors. Our study’s findings support previous research by showing that age, education level, marital status, place of residence, types of chronic diseases, and frailty status, are all associated with fall risk [34]. As individuals age, their body function declines, making them more susceptible to diseases and increasing their risk of falls [35]. Place of residence and marital status are important components of social support, which has a strong correlation with fall risk. Therefore, elderly individuals with different places of residence and marital statuses may have varying levels of fall risk, and those with higher social support tend to have lower fall risk [36]. Additionally, individuals with higher education levels demonstrate better medication compliance, prioritize maintaining their health, and reduce their risk of falls [37]. In addition, increased fall risk is associated with increased chronic diseases. Chronic diseases can affect the physical function, body pain, general health, vitality, and mental health of the elderly. The more the number of chronic diseases, the worse the general health and quality of life of the elderly [38, 39]. Compared with disease-specific fall injury intervention, health management based on physical function may be more effective in preventing and reducing fall injuries. Furthermore, physical, psychological, and social frailty of the elderly all harm falls in the elderly. These results are consistent with the results of most previous studies [40, 41]. In addition to the physiological factors, the psychological factors of the elderly should be given equal attention. Previous studies have reported that physical, psychological, and social factors lead to decreased activity in the elderly, which in turn reduces the confidence and ability to perform activities and leads to the fear of falling [42]. Previous studies have demonstrated that the fear of falling is associated with a higher risk of falls among the elderly [43, 44]. Consequently, it is crucial to prioritize the mental well-being of older individuals and promote their engagement in moderate exercise, as this approach may yield positive outcomes in fall prevention.

The results of this study demonstrate a positive correlation between oral frailty and fall risk, which aligns with the findings of Tatsuo Yamamoto [45]. This could be attributed to the impact of oral weakness on jaw position, subsequently affecting head and neck posture, overall body posture, and balance, ultimately leading to falls [46]. Addressing individual oral health issues becomes crucial in mitigating the risk of falls among the elderly. Additionally, Our study revealed a positive association between nutrition and fall risk. A meta-analysis study involving 9 510 subjects indicated that individuals who were malnourished or at risk of malnutrition had a 45% higher risk of experiencing at least one fall compared to their well-nourished counterparts [47]. The synthesis of muscle mass requires specific nutrients, and maintaining a dynamic balance between protein supply and demand is crucial. If this balance is disrupted for an extended period, it can lead to a decline in muscle mass and strength, ultimately contributing to sarcopenia in older adults [48] and increasing the risk of falls. In conclusion, both oral frailty and nutrition are factors that influence the risk of falls. It is recommended that caregivers regularly assess oral health and nutrition, encourage individuals to prioritize oral care and exercise, and improve overall nutrition. These measures can help reduce the risk of falls and enhance the quality of life for the elderly.

The findings indicate that oral frailty has a direct impact on the fall risk of the elderly and also indirectly affects it through nutrition. Previous studies [49–51] have mainly focused on the relationship between oral frailty, nutrition, and fall risk individually. This study contributes to the existing research by conducting a mediation analysis to explore the relationship between oral frailty, nutrition, and fall risk. In terms of direct effects, oral frailty can directly predict the risk of falls. Besides the previously mentioned impact of oral frailty on fall risk through occlusal support, inflammatory mechanisms can also influence the balance of the elderly [52]. Local inflammation caused by oral frailty can result in a systemic inflammatory response, leading to muscle mass loss, increased incidence of sarcopenia, and falls [53, 54]. Moreover, oral frailty can indirectly increase fall risk through its impact on nutritional status. Elderly individuals with oral frailty often reduce their intake of harder foods like meat, fruits, and vegetables, which are important sources of vitamins, proteins, and trace elements. This deficiency in key nutrients can lead to malnutrition, ultimately contributing to sarcopenia and falls [55, 56]. Therefore, it is crucial to prioritize oral health, monitor food intake, and maintain a balanced diet to protect the elderly.

### Limitations of the research

There are some limitations to consider in this study. Firstly, the study sample consists of robust, community-dwelling older adults, which limits the generalisability of the study findings. Secondly, our study design was a cross-sectional study. Thirdly, Fall itself is a complex outcome caused by many factors. Although this study used the Tilburg frailty index to comprehensively investigate some physical, psychological, and social information of the participants, some of the assessments did not seem to accurately evaluate the state of the subjects (such as grip strength). All of these were potential sources of bias. A longitudinal study with some more representative samples may be required to establish the relationship between oral frailty, nutrition, and fall risk.

## Conclusion

Preventing falls among the elderly is crucial for promoting a healthy aging society. This study highlights a significant risk of falls among elderly individuals in the community in China. Furthermore, it suggests that oral frailty and nutrition play a significant role in increasing the likelihood of falls, with nutrition partially mediating the relationship between oral frailty and fall risk. These findings offer a novel intervention approach to mitigate fall risk in individuals with oral frailty and provide valuable insights into understanding the mechanisms underlying fall risk associated with oral frailty.

In the future, it is recommended that medical staff enhance the assessment of oral frailty and nutrition among this population. Additionally, providing appropriate health education and support can help improve the oral frailty and nutrition of patients. This, in turn, can effectively reduce the risk of falls and enhance the quality of life for the elderly.

## Data Availability

The data used to support the findings of this study are available from the corresponding author upon reasonable request.

## Abbreviations

OFI: 8 Oral Frailty Index-8
MNA-SF: Short form mini nutritional assessment
SIB: Stay Independent Brochure
STEADI: Stopping Elderly Accidents, Deaths & Injuries Toolkit
OHAT: Oral Health Assessment Tool
TFI: Tilburg frailty index
